# Right heart failure before LVAD implantation predicts right heart failure after LVAD implantation – is it that easy?

**DOI:** 10.1186/s13019-020-01150-x

**Published:** 2020-05-25

**Authors:** Tobias Wagner, Alexander M. Bernhardt, Christina Magnussen, Hermann Reichenspurner, Stefan Blankenberg, Hanno Grahn

**Affiliations:** 1 Department of General and Interventional Cardiology, University Heart Center Hamburg, Martinistr. 52, D-20246 Hamburg, Germany; 2 Department of Cardiovascular Surgery, University Heart Center Hamburg, Hamburg, Germany; 3 German Center for Cardiovascular Research (DZHK), partner site Hamburg/Kiel/Luebeck, Hamburg, Germany

**Keywords:** LVAD, Ventricular assist device, Continuous flow, Right heart failure

## Abstract

**Background:**

Right heart failure (RHF) after left ventricular assist device (LVAD) implantation is common and associated with worse outcome. Prediction of RHF remains challenging. Our study aims to assess predictors of RHF focusing on clinical manifestations.

**Methods:**

We retrospectively analyzed clinical, echocardiographic and hemodynamic parameters of 112 patients undergoing LVAD implantation. Pre-operative, early (ERHF, day 7 and 14) and late postoperative RHF (LRHF, after 1, 3, 6 and 12 months) were assessed.

**Results:**

In the total study population (87.5% men, mean age 55 years), early RHF was frequent (47% on day 7 and 30% on day 14). Prevalence of late RHF and death from RHF was high after 3, 6 and 12 months (23, 24 and 17%). Pre-existing RHF was only associated with early RHF and persistent, but not for new onset late RHF. Early RHF was associated with lower INTERMACS level (*p* < 0.001), higher pulmonary vascular resistance (*p* = 0.046) and CVP/PAWP quotient (*p* = 0.011), higher bilirubin (*p* = 0.038) and creatinine (*p* = 0.013). LRHF was associated with creatinine (*p* = 0.006), urea (*p* = 0.012) and load adaption index (*p* = 0.007). Binary logistic regression models identified no single risk factors. Comparing the predictive value of regression models with a model of three clinical findings (INTERMACS level, age and pre-operative RHF) did not reveal differences in RHF.

**Conclusions:**

RHF before LVAD implantation enhances the risk of early RHF and persistent late RHF, but not for new onset late RHF, supporting the hypothesis of differences in the etiology. Echocardiographic or hemodynamic parameters did not show a predictive value for new onset late RHF. Similar predictive value of clinical findings and statistic models of risk factors suggest that a clinical evaluation is equally matched to predict RHF.

## Background

Due to the lack of heart donors, mechanical assist device support has become an important alternative to heart transplantation in care among patients with advanced heart failure. Despite advances in technology and greater familiarity with patient management, overall survival remains essentially unchanged since 2013 at 80% after 1 year and 70% after 2 years [1].

Right heart failure (RHF) is one of the most common complications after continuous flow left ventricular assist device (LVAD) implantation with high impact on outcome [2, 3]. Therefore, it is crucial to identify preoperative predictors of RHF post-LVAD implant.

Many attempts were done to predict RHF after LVAD implantation. These approaches include echocardiographic parameters like TAPSE. Besides, parameters derived from right heart catheterization like central venous pressure (CVP) and laboratory parameters like bilirubin or creatinine were used. All these attempts failed to predict RHF accurately [4, 5].

In addition, combinations of parameters like load adaptation index of the right ventricle (LAI) and tricuspid valve velocity time integral (VTI_TR_) [6] or complex quantitative scores [7, 8] predominantly derived from monocentric patient populations try to predict RHF. The usefulness of RHF risk prediction models is limited primarily because the patients in these cohorts were supported with earlier-generation pulsatile-flow devices, which are not comparable to the current continuous-flow LVAD devices. Modest discrimination in the derivation cohort lead to moderate predictive values. A recent meta-analysis [9] of observational studies concludes that the available tools are not sufficient to identify patients with high risk. Further, definition of RHF is inconsistent which limits the comparison of research results.

By analyzing epidemiological aspects of RHF *before* LVAD implantation, we aimed to show their impact on early and late RHF *after* LVAD implantation. We refer to and focus on the INTERMACS definition of RHF [10] that is based on signs of high CVP and clinical or laboratory signs of congestion. In our cohort of 112 patients, we analyzed the prevalence and incidence of RHF over time after LVAD implantation. Further, we assessed risk factors and the impact of pre-operative and post-operative RHF on survival. Finally, we compared the predictive value of risk factors identified by a regression model and a risk stratification including only demographic and clinical parameters.

## Methods

### Patients and devices

The screening population consisted of all subjects ≥18 years of age undergoing elective or emergency LVAD implantations between January 2009 and May 2017 at University Heart Center Hamburg. A total of 132 subjects met the screening criteria. We excluded all patients that did not receive a continuous flow device of the HVAD™ type from Heartware® (*n* = 3). Furthermore, we excluded patients who additionally received a right ventricular assist device (RVAD) for permanent biventricular support (BiVAD, *n* = 14) and patients with the need for chronic hemodialysis before LVAD implantation (n = 1). Two cases with lost-to-follow up < 14 days after surgery were excluded.

### Definition of RHF

Criteria of RHF were adapted from INTERMACS definition [10] requiring 7 days of support and consisting of two criteria:

1. Documentation of elevated CVP by direct measurement (CVP or RAP > 16 mmHg) or dilated inferior vena cava with absence of inspiratory variation or elevated jugular venous distension.

2. Manifestations of elevated central venous pressure characterized by peripheral edema (> 2 either new or unresolved), presence of ascites or palpable hepatomegaly (physical examination or diagnostic imaging), or laboratory evidence of hepatic (total bilirubin > 2.0 mg/dl) or renal dysfunction (creatinine > 2.0 mg/dl).

Early RHF and late RHF were defined by fulfilling these criteria 7–14 days and > 14 days after surgery, respectively. Pre-operative RHF was defined by the same criteria at the day of surgery.

### Data collection and follow-up

Clinical and demographic data and variables assessed by pre-operative echocardiography, laboratory and right heart catheterization were retrospectively extracted from patient records. Presence of RHF 7, 14 and 30 days as well as 3, 6 and 12 months after surgery were independently checked and validated by two physicians trained in cardiovascular medicine. Conflicting results were solved by reviewing the patients’ record again and making a consensus decision. Besides presence of RHF, also a combined outcome of RHF or death due to RHF after 30 days and 1 year was defined. For long-term outcome, at least one follow-up ≥30 days after surgery was required. In case of death or loss to follow-up, the last observation was carried forward.

### Statistical analysis

Categorical data were summarized descriptively by frequencies along with the associated percentages and were compared by Chi-Squared or Fisher’s Exact Test dependent on variable characteristics and distribution. Continuous variables were summarized by mean and standard deviation (SD) and were compared by Student T-test.

Risk factors for early RHF and late RHF were analyzed using a multiple logistic regression model. Covariates were selected by univariate factor analyses. A variable was included if *p* < 0.1 in at least one analysis. Backward selection was performed with *p* < 0.3. Model fit was evaluated by Hosmer Lemeshow Test. Nagelkerke‘s R^2^ was reported. Kaplan-Meier curves were used to analyze survival and were compared by log-rank test.

A 5% level of significance (two-sided) was used for any statistical test. Descriptive, interferential and survival statistics were performed by IBM SPSS, Version 24 for Windows. Missing data were imputed by “missForest” package for R [11] to increase the yield of logistic regression and allow backward regression. To compare the areas under the curve (AUC), the “pROC” packagefor R to perform De Long tests was used [12].

## Results

### Baseline characteristics and pre-operative RHF

Patients had a mean age of 55 years (SD 13), 12.5% were female. Pre-operative RHF was present in 72 cases (64.3%). Two thirds of devices were implanted as bridge to transplant (BTT). In half of the patients, dilated cardiomyopathy was the underlying disease, while 34.8% of patients suffered from ischemic cardiomyopathy. Almost all patients had a left ventricular ejection fraction (EF) < 30%. Patients with baseline EF > 30% were treated within the context of fatal heart failure after cardiac surgery. Apart from highly reduced EF, moderate or severe mitral valve regurgitation (46.5%) and tricuspid valve regurgitation (40.2%) were frequent.

Patients with pre-operative RHF had lower age (*p* = 0.007), a higher CVP/PAWP quotient (*p* = 0.026) and CVP (*p* = 0.032) than patients without pre-operative RHF. They had an infection more frequently, lower hemoglobin (*p* = 0.003) and a lower INTERMACS level (*p* < 0.001). There were no differences in parameters of the echocardiographic assessment, in the underlying disease and the indication for LVAD implantation (Table 1).

**Table 1:** Baseline Data

Baseline patient characteristics				
	Whole group (*n* = 112)	RHF pre OP (*n* = 72)	No RHF pre OP (*n* = 40)	***p***-value
Age	55.3 (12.5)	53.0 (12.4)	59.6 (11.7)	0.007
Male	98 (87.5)	63 (87.5)	35 (87.5)	1.000
BMI	26.5 (4.5)	27.0 (3.8)	26.1 (5.0)	0.400
**INTERMACS**				
▪ 1–2	49 (43.8)	42 (58.3)	7 (17.5)	
▪ 3–4	40 (35.7)	19 (26.4)	21 (52.5)	
▪ 5–7	23 (20.5)	11 (15.3)	12 (30.0)	< 0.001
**Indication for LVAD therapy**				
▪ BTT	76 (67.9)	50 (69.4)	26 (65.0)	
▪ DT	31 (27.7)	17 (23.6)	14 (35.0)	
▪ BTR	3 (2.7)	3 (4.3)	0 (0.0)	
▪ Emergency	2 (1.8)	2 (2.8)	0 (0.0)	0.379^#^
**Underlying disease**				
▪ Ischaemic cardiomyopathy	39 (34.8)	23 (31.9)	16 (40.0)	
▪ Dilated cardiomyopathy	62 (55.4)	39 (54.2)	23 (57.5)	
▪ Acute myocarditis	4 (3.6)	3 (4.2)	1 (2.5)	
▪ Postcardiotomy syndrome	4 (3.6)	4 (5.6)	0 (0.0)	
▪ Other/not known	3 (2.7)	3 (4.2)	0 (0.0)	0.834^##^
**Echocardiography**				
EF < 30%^###^	107 (95.5)	69 (95.8)	38 (95.0)	0.838
Moderate or severe mitral valve regurgitation	52 (46.5)	33 (45.8)	19 (47.5)	0.865
Moderate or severe tricuspid valve regurgitation	45 (40.2)	33 (45.8)	12 (30.0)	0.101
TAPSE	14.6 (4.1)	14.6 (4.4)	14.6 (3.5)	0.968
V. cava inferior diameter	19.4 (4.1)	19.4 (4.0)	19.2 (4.4)	0.758
Load adaption index (LAI)	26.4 (9.1)	25.5 (8.9)	28.0 (9.5)	0.161
**Right Heart Catheter**				
PVR [Dyn*s/cm^5]	227.6 (154.9)	237.2 (186.5)	216.8 (111.0)	0.599
CI [L/min*m^−2^]	1.9 (0.5)	2.0 (0.6)	1.8 (0.4)	0.133
PAWP [mmHg]	23.9 (10.3)	24.2 (9.3)	23.7 (11.4)	0.847
mPAP [mmHg]	31.4 (12.2)	32.8 (11.4)	30.0 (13.0)	0.339
CVP [mmHg]	10.8 (6.4)	12.4 (6.6)	9.1 (5.8)	0.032
CVP/PAWP	0.47 (0.25)	0.54 (0.28)	0.40 (0.18)	0.026
**Laboratory results**				
Hb [g/dL]	11.3 (2.3)	10.8 (2.1)	12.1 (2.3)	0.003
Leucocytes [Mrd/L]	9.6 (5.1)	10.2 (5.0)	8.6 (5.0)	0.113
Thrombocytes [Mrd/L]	198.8 (91.2)	193.0 (90.6)	209.3 (90.4)	0.367
Total Bilirubin [mg/dL]	1.1 (1.1)	1.3 (1.3)	0.9 (0.5)	0.018
Urea [mg/dL]	35.2 (19.2)	37.3 (21.4)	31.5 (21.4)	0.081
Creatinine [mg/dL]	1.8 (0.9)	1.9 (1.0)	1.5 (0.6)	0.020
GOT [U/L]	185.1 (525.8)	65.9 (114.9)	251.3 (642.2)	0.002
GPT [U/L]	145.7 (384.3)	193.7 (461.2)	59.3 (145.3)	0.025
GGT [U/L]	137.5 (100.0)	145.7 (109.7)	122.7 (78.7)	0.244
CRP [mg/L]	36.3 (50.7)	44.4 (57.4)	21.8 (31.3)	0.008
INR	1.4 (0.5)	1.3 (0.4)	1.5 (0.7)	0.222

Values are presented as mean (SD) and compared by Student T-test. Categorical data a shown as n (%) and groups were compared by Chi square test. ^#^BTT vs. DT. ^##^Ischaemic vs. dilatative. ^###^In a few patients initial EF was > 35% (mainly intra-operative decompensation)

### Incidence of early and late RHF and impact on mortality

**Fig 1:**
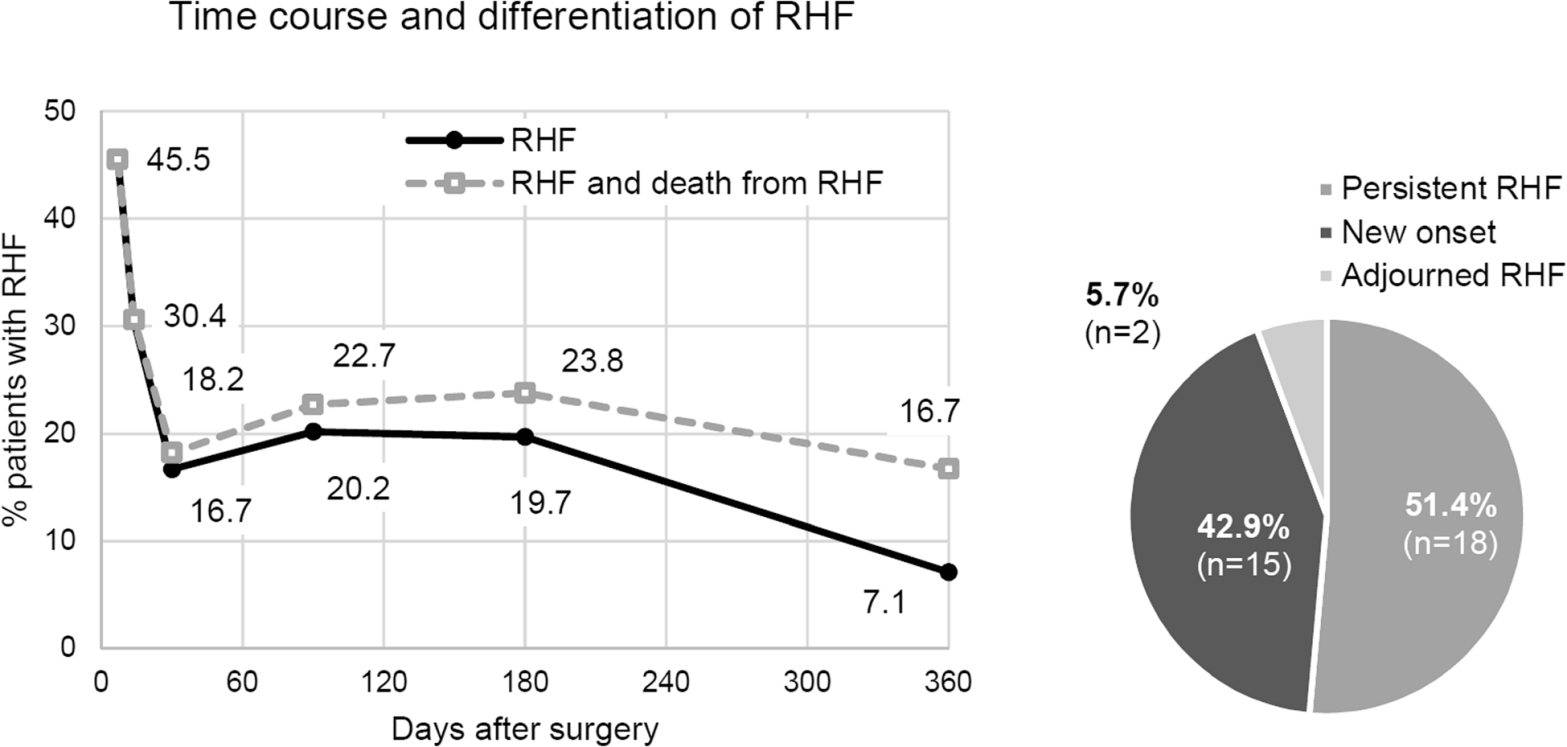
Time course and differentiation of RHF

Fourteen days after LVAD implantation, 30% of patients suffered from RHF. A preliminary low prevalence (16.7%) was reached 30 days after surgery. Afterwards the prevalence increased and the share of patients who died due to RHF grew. Only half of the patients with late RHF were permanently affected by RHF. The other half had new onset RHF. Within the first 6 months, RHF was the most common cause of death (31.4% of deaths). In contrast, only 5% of deaths were classified as caused by RHF afterwards while sepsis and major bleeding became more frequent causes of death (Fig. 1).

### Impact of pre-operative RHF on post-operative RHF and survival

Pre-operative RHF was a strong risk factor for ERHF (OR = 5.0, 95% CI 1.75–14.26, *p* < 0.001) and persistent RHF (OR = 3.98, 95% CI 1.07–14.84, *p* = 0.030), but not for new onset LRHF (OR = 0.69, 95% CI 0.23–2.10, *p* = 0.518). The same pattern was found for the combined outcomes for early RHF or death from RHF (OR 4.45, 95% CI 1.20–16.49, *p* = 0.018) and late RHF or death from RHF (OR = 0.94, 95% CI 0.30–2.92, *p* = 0.919, Table 2). One year after LVAD implantation, there was no evidence for a difference in survival (*p* = 0.299) between patients with and without pre-operative RHF. Nevertheless, pre-operative RHF was associated with a lower survival rate (*p* = 0.020) up to 14 days after surgery. Afterwards, the impact of pre-operative RHF diminished.

**Table 2:** Odds Ratios in case of pre-operative RHF

Odds Ratios in case of pre-operative RHF	OR	95% CI	P
RHF early	5.00	1.75–14.26	< 0.001
RHF late	2.06	0.85–5.00	0.108
- RHF late persistent	3.98	1.07–14.84	0.030
- RHF late new	0.69	0.23–2.10	0.518
Combined outcome 30 days	4.45	1.20–16.49	0.018
Combined outcome 360 days	0.94	0.30–2.92	0.919

**Fig. 2:**
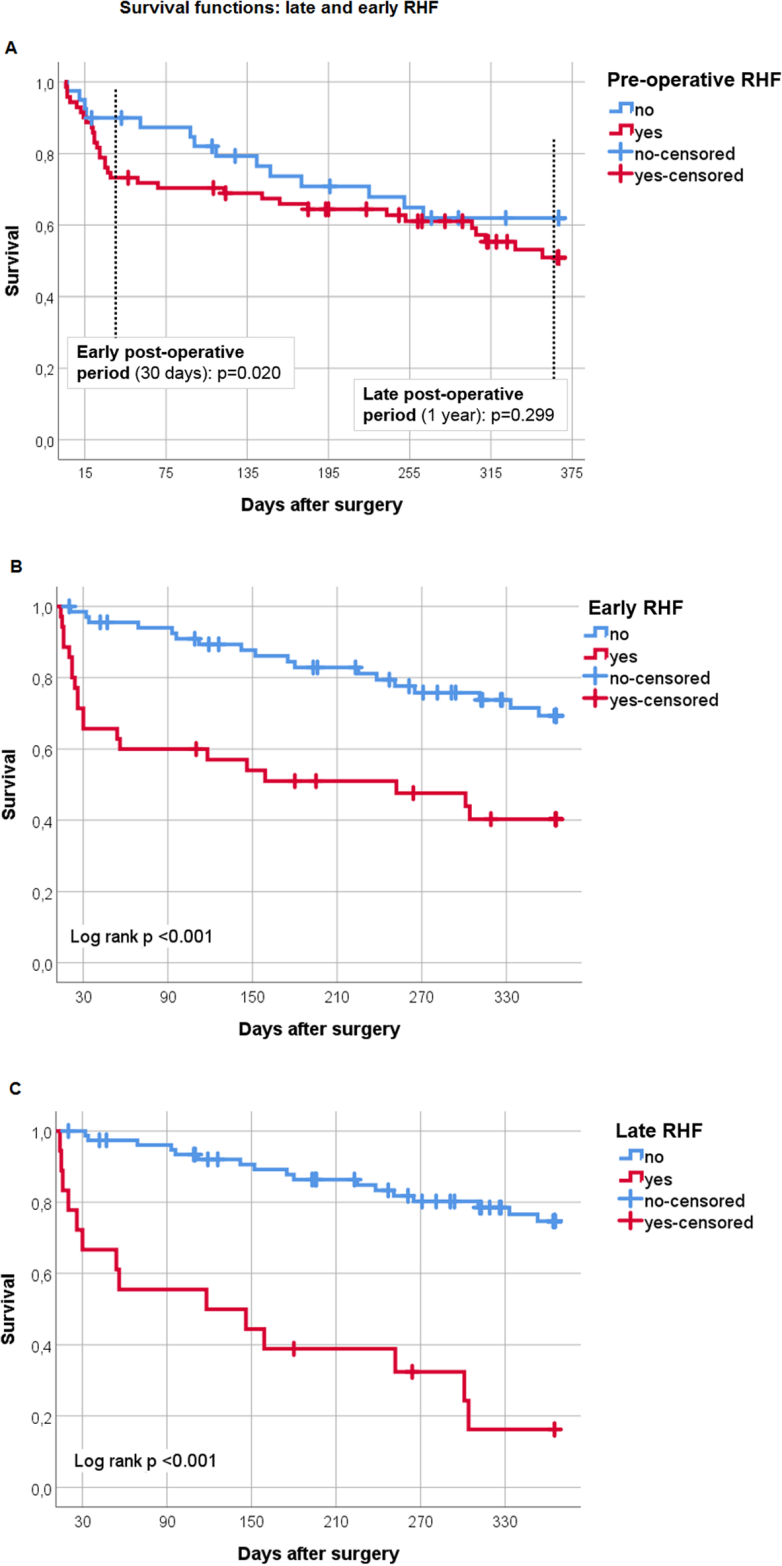
Survival functions: late and early RHF

Survival of patients with early RHF was significant lower (*p* < 0.001) than of patients without early RHF. Differences between the survival curves developed within the first 2 weeks and remained stable. Survival of patients with ongoing early RHF, recurrent or new onset late RHF was worse (*p* < 0.001) compared to patients without post-operative RHF or RHF only in the early post-operative period (Fig. 2).

### Risk factors for RHF

Patients with early RHF were younger (*p* = 0.023), classified into a lower INTERMACS level (*p* < 0.001), had a higher CVP/PAWP quotient (*p* = 0.011), lower load adaption index (*p* = 0.019) and higher urea (*p* = 0.010) and creatinine (*p* = 0.013). Regarding late RHF, associations were obvious for creatinine (*p* = 0.006) and urea (*p* = 0.012) and load adaption index (*p* = 0.007, Supplemental Table 1). The combined endpoints considering patients with RHF and patients who died from RHF 1 month respectively 1 year after surgery principally revealed the same risk factors (Supplemental Table 2).

Early RHF was independently predicted by lower age (OR = 0.93, *p* = 0.05), male gender (OR = 2.49, *p* = 0.04), low TAPSE (OR = 1.35, *p* < 0.01), high creatinine (OR = 6.48, p < 0.01) and high CVP (OR = 1.12, *p* = 0.08). Furthermore, GOT (OR = 0.99, *p* = 0.07) and GPT (OR = 1.01, p = 0.08) remained in the model.

Late RHF was associated with high creatinine (OR = 7.13, p = 0.08), low hemoglobin concentration (OR = 2.81, *p* = 0.06), low thrombocytes (OR = 1.01, *p* = 0.28) and low VTI_TR_ (OR = 1.07, *p* = 0.03) as independent risk factors (Table 3).

**Table 3:** Independent risk factors for early and late RHF (multiple logistic regression models)

Early RHF (R^2^ = 0.502)					
	B	S.E.	p	OR	95% CI
High age	−0.07	0.04	0.05	0.93	0.87–1.00
Male gender	2.53	1.24	0.04	12.49	1.11–140.97
Low TAPSE [mm]	0.30	0.12	< 0.01	1.35	1.08–1.70
High Creatinine [mg/dl]	1.87	0.71	< 0.01	6.48	1.61–26.13
High GOT [U/L]	−0.01	0.01	0.07	0.99	0.98–1.00
High GPT [U/L]	0.01	0.01	0.08	1.01	1.00–1.02
High CVP [mmHg]	0.11	0.06	0.08	1.12	0.99–1.26
**Late RHF** (R^2^ = 0.655)					
	B	S.E.	p	OR	95% CI
High Creatinine [mg/dL]	1.96	1.11	0.08	7.13	0.80–63.24
Low Hb [g/dL]	1.04	0.56	0.06	2.81	0.94–8.43
Low Thrombocytes [Mrd/L]	0.01	0.01	0.28	1.01	0.99–1.03
Low VTI der TK [cm]	0.07	0.03	0.03	1.07	1.01–1.14

### Comparison of the predictive value of clinical and technical risk factors

**Fig. 3:**
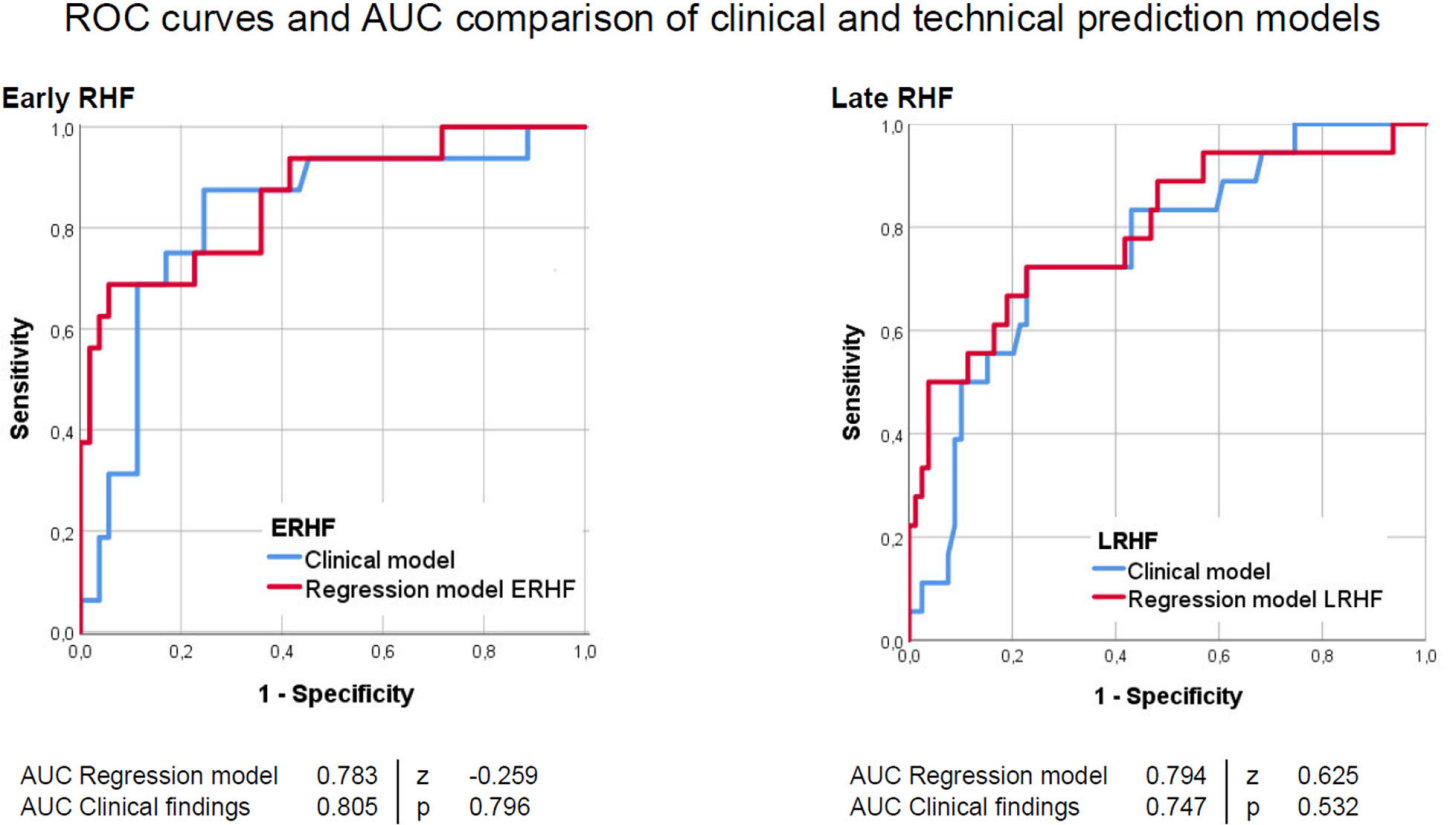
ROC curves and AUC comparison of clinical and technical prediction models

Finally, we compared the predictive value of the binary logistic regression models with a model of three clinical findings (INTERMACS level, age and presence of pre-operative RHF) accordingly. The models’ AUC ranged from 0.747 to 0.805 (Fig. 3). De Long tests did not reveal significant difference between the technical and the clinical model (Table 4).

**Table 4:** Compare of clinical and technical prediction models

Outcome	AUC Clinical model	AUC Technical model	p
Early RHF	0.805	0.783	0.796
Late RHF	0.747	0.794	0.532
Combined outcome 30 days	0.730	0.699	0.651
Combined outcome 360 days	0.618	0.703	0.291

## Discussion

RHF after LVAD implantation can appear as short-term as well as long-term complication. We show that both early RHF and late RHF are common and have a negative impact on patient outcome. RHF before LVAD implantation was associated with a higher risk of early RHF and persistent late RHF, but not for new onset late RHF. Single echocardiographic, laboratory and hemodynamic parameters did not strongly predict new onset late RHF. In our cohort, similar predictive value of clinical findings and statistic models of risk factors highlight clinical evaluation to predict RHF.

Especially the prevalence of late and chronic RHF becomes increasingly important due to the growing number of patients with long-term LVAD support. Hitherto, no discrete factor with adequate discrimination and satisfying reliability was found. In a recent meta-analysis, the only predictors strongly associated with RHF were INR and CVP. Reduced cardiac index and high pulmonary vascular resistance were the main moderators of the relationship between INR and RHF [9].

The vast majority of studies focus on patients that suffer from RHF early post LVAD implantation in order to identify patients needing a temporary RVAD implantation [2, 8, 13–16]. Risk stratification for late RHF was investigated only in small clinical cohorts and is therefore poorly understood. The incidence of LRHF is widespread dependent on the definition (11–45%) [17–19]. In a study of Kapelios et al. [17], only creatinine and systemic vascular resistance had a predictive value for late RHF, but the parameters were not analyzed in a multiple regression model. In parallel, in our study, serum creatinine was the variable with the highest odds ratio regarding LRHF and death 1 month and 1 year after surgery.

Takeda et al. [18] assessed the prevalence and risk factors of late RHF in 293 LVAD patients. Similar to our approach, detection of late RHF was based on typical signs and symptoms including edema, weight gain, ascites and jugular vein distention, but only re-hospitalized patients were taken into account rendering the prevalence much smaller than in our study. In contrast to our findings, late RHF did not affect survival during LVAD support. However, the authors found that late RHF was associated with worse overall outcomes in their small bridge-to-transplant population. Like in our study, pre-operative echocardiographic or hemodynamic parameters did not show a clear predictive value for onset of late RHF [18].

Transpulmonary gradient (diastolic and/or mean) and corresponding pulmonary resistance are surrogates for right ventricular afterload and determine if a patient is eligible for transplant. They might also predict the risk for postoperative RHF and/or mortality in LVAD recipients [20]. LVAD therapy might be able to qualify patients for transplant who were not eligible due to preliminary existing pulmonary hypertension by lowering pulmonary artery pressure. In our study, we could not show an impact of these parameters on RHF and on RHF associated mortality.

Irrespective of the underlying mechanism, knowledge about whether or not and when a patient will develop RHF after LVAD implantation would enable clinicians to individually determine patient-adjusted strategies (BTT, DT, BiVAD or to refrain from LVAD implantation).

Strategies to optimize right ventricular function before LVAD implantation are limited to patients with stable heart failure. Nevertheless, the benefit of prophylactic therapy with inotropic agents, pulmonary vasodilators and volume management by hemodialysis guided by CVP in order to prevent RHF is not clear [9].

There are several definitions of RHF differing in criteria, time scope and severity. In most studies, RHF is defined as the requirement of unplanned RVAD implantation, nitric oxide or iloprost inhalation for more than 48 h or intravenous inotropic therapy for more than 14 days after surgery [9]. For evaluation of late RHF, this definition is unemployable. In addition, a uniform definition of early (acute, post-operative) and late (mainly chronic) RHF is essential to compare results of research. Here we adapted the INTERMACS classification [10] because it is suitable both for early RHF and late RHF. Furthermore, the criteria are easy to apply and allows clinical decisions in a comprehensible manner. To our knowledge, this is the first study in which pre-operative, early and late RHF is described according to a uniform definition based on clinical signs and the relationship of RHF at different points in the patient’s history is illustrated.

Here we could show that the predictive value of a model including three clinical findings (INTERMACS level, age and pre-operative RHF according to the clinical definition) is equal to technical regression models.

### Limitations

The main limitations of our study is the retrospective, mono-centric study design, and the sample size. We assessed a broad, but unquestionably limited set of parameters. Since the model development sample was used for model validation, generalization of results should be applied with caution. We excluded all patients who did not receive an HVAD and patients who underwent primary long-term BiVAD implantation. This might constitute as a selection bias, but it renders relevant homogeneity to our cohort.

We chose a ‘broad’ clinical definition of RHF resulting in a high prevalence of RHF compared to other studies. As intra-operative variables such as volume, inotropic and transfusion management were not considered, their influence on outcome remains undetected.

Hemodynamic parameters were not re-assessed in a regularly unless necessary for transplant evaluation. Consequently, we cannot report the impact of LVAD therapy on parameters of pulmonary hypertension.

## Conclusions

Our results show that RHF before LVAD implantation is associated with a high rate of ERHF after surgery and is a predictor for worse outcome. Assessment of pre-operative clinical findings can help to identify patients at risk that probably profit from aggressive pre-operative optimization of right heart function gained by diuretics, inotropes or hemodialysis.

Late RHF was only partly associated with pre-operative or early RHF indicating that a regular re-evaluation of the right ventricular function is mandatory and could be helpful to prevent new onset of late RHF. This predictive model should be validated in a bigger LVAD cohort. In the age of growing importance of biomarkers and imaging modalities the importance of clinical findings should not be underestimated.

## Supplementary information

**Additional file 1: Supplemental Table 1**. Risk factors for early and late RHF.

**Additional file 2: Supplemental Table 2**. Risk factors for RHF and death from RHF after one month and one year.

## Data Availability

The datasets used for the this study are available from the corresponding authors on reasonable request.

## Abbreviations

AUC: Area under the curve
BiVAD: Biventricular assist device
BTR: Bridge to recovery
BTT: Bridge to transplant
CI: Cardiac index
CVP: Central venous pressure
DT: Destination therapy
INTERMACS: Interagency Registry for Mechanically Assisted Circulatory Support
EF: Ejection fraction
GGT: Gamma-glutamyltransferase
GOT: Glutamic oxaloacetic transaminase
GPT: Glutamate-pyruvate transaminase
Hb: Hemoglobin
INR: International Normalized Ratio
LAI: Load adaption index of the right ventricle
LVAD: Continuous flow left ventricular assist device
mPAP: Mean pulmonary arterial pressure
PAWP: Pulmonary arterial wedge pressure
PVR: Pulmonary vascular resistance
RHF: Right heart failure
RVAD: Right ventricular assist device
TAPSE: Tricuspid annular plane systolic excursion
VTI_TR_: Tricuspid valve velocity time integral
