# Supplementary material for: Right heart failure before LVAD implantation predicts right heart failure after LVAD implantation – is it that easy?
Source: J Cardiothorac Surg. 2020 May 25;15:113. doi: 10.1186/s13019-020-01150-x (PMC7249428; doi:10.1186/s13019-020-01150-x)
Supplement: Supplementary file 1 — Additional file 1: Supplemental Table 1. Risk factors for early and late RHF. [file 13019_2020_1150_MOESM1_ESM.docx]

Supplemental Table 1 Risk factors for early and late RHF

|  | **EARLY RHF** | | | **LATE RHF** | | |
| --- | --- | --- | --- | --- | --- | --- |
|  | **Early RHF** (n=35) | **No early RHF** (n=77) | p | **Late RHF** (n=35) | **No late RHF** (n=62) | p |
| Age | 51.4 (13.5) | 57.1 (11.7) | 0.023 | 53.7 (13.2) | 55.1 (12.8) | 0.614 |
| Male | 30 (85.7) | 68 (88.3) | 0.761 | 30 (78.9) | 54 (91.5) | 0.853 |
| **INTERMACS** |  |  |  |  |  |  |
| ▪ 1-2 | 24 (68.6) | 25 (32.5) |  | 20 (57.1) | 19 (30.7) |  |
| ▪ 3-4 | 10 (28.6) | 30 (39.0) |  | 10 (28.6) | 27 (43.5) |  |
| ▪ 5-7 | 1 (2.8) | 22 (28.5) | <0.001 | 5 (14.3) | 16 (25.8) | 0.188 |
| **Indication for LVAD therapy** |  |  |  |  |  |  |
| ▪ BTT | 28 (80.0) | 48 (65.8) |  | 25 (75.8) | 42 (70.0) |  |
| ▪ DT | 6 (17.1) | 25 (34.2) | 0.109 | 8 (24.2) | 18 (30.0) | 0.654 |
| **Underlying disease** |  |  |  |  |  |  |
| ▪ Ischaemic | 15 (42.9) | 24 (31.2) |  | 17 (48.6) | 18 (29.0) |  |
| ▪ Dilatative | 16 (45.7) | 46 (59.7) | 0.192 | 14 (40.0) | 39 (62.9) | 0.090 |
| **Echocardiography** |  |  |  |  |  |  |
| EF <30% | 34 (97.1) | 73 (94.8) | 1.000 | 34 (97.1) | 58 (93.5) | 1.000 |
| Moderate or severe mitral valve regurgitation | 15 (42.9) | 33 (42.9) | 0.482 | 14 (40.0) | 31 (50.0) | 0.509 |
| Moderate or severe Tricuspid valve regurgitation | 12 (34.3) | 28 (36.4) | 0.817 | 14 (40.0) | 24 (38.7) | 1.000 |
| TAPSE [mm] | 14.1 (5.8) | 14.6 (4.4) | 0.863 | 13.4 (4.9) | 15.1 (4.8) | 0.164 |
| VCI diameter | 19.3 (5.3) | 19.5 (4.8) | 0.693 | 18.5 (5.3) | 19.6 (4.5) | 0.364 |
| LAI | 21.9 (9.0) | 27.2 (10.4) | 0.019 | 21.7 (9.2) | 27.7 (10.8) | 0.007 |
| **Right heart catheter** |  |  |  |  |  |  |
| PVR [Dyn*s/cm^5^] | 168 (103) | 258 (175) | 0.046 | 183 (143) | 264 (176) | 0.081 |
| CI [L/min*m^2^] | 2.1 (0.5) | 1.9 (0.5) | 0.098 | 2.1 (0.5) | 1.9 (0.5) | 0.441 |
| PAWP [mmHg] | 26.9 (9.6) | 22.9 (10.5) | 0.180 | 22.8 (9.8) | 24.2 (11.0) | 0.628 |
| mPAP [mmHg] | 33.4 (11.2) | 31.7 (12.0) | 0.447 | 30.5 (10.9) | 33.2 (12.4) | 0.321 |
| CVP [mmHg] | 13.6 (5.4) | 10.0 (6.6) | 0.054 | 11.6 (6.1) | 10.3 (6.8) | 0.447 |
| CVP/PAWP | 0.60 (0.33) | 0.43 (0.20) | 0.011 | 0.53 (0.26) | 0.44 (0.24) | 0.163 |
| **Laboratory** |  |  |  |  |  |  |
| Hb [g/dL] | 10.5 (2.0) | 11.6 (2.3) | 0.013 | 10.7 (2.0) | 11.8 (2.4) | 0.024 |
| Leucocytes [Mrd/L] | 9.9 (5.1) | 9.5 (5.1) | 0.733 | 8.7 (4.8) | 9.7 (4.6) | 0.312 |
| Thrombocytes [Mrd/L] | 179 (93) | 208 (90) | 0.126 | 184 (85) | 217 (91) | 0.080 |
| Total bilirubin [mg/dL] | 1.6 (1.7) | 0.9 (0.6) | 0.038 | 1.5 (1.7) | 0.9 (0.5) | 0.050 |
| Urea [mg/dL] | 42.4 (21.9) | 32.1 (17.4) | 0.010 | 40.3 (20.1) | 30.2 (17.2) | 0.012 |
| Creatinine [mg/dl] | 2.1 (1.1) | 1.6 (0.7) | 0.013 | 2.1 (1.0) | 1.5 (0.7) | 0.006 |
| GOT [U/L] | 153.6 (270.6) | 200.7 (602.9) | 0.669 | 104.3 (248.9) | 87.8 (123.3) | 0.669 |
| GPT [U/L] | 147.2 (353.5) | 146.0 (402.3) | 0.988 | 121.7 (346.9) | 74.2 (131.6) | 0.441 |
| GGT [U/L] | 153.1 (106.7) | 129.0 (100.8) | 0.267 | 142.1 (115.5) | 117.9 (79.5) | 0.236 |
| CRP [mg/L] | 54.7 (64.4) | 27.2 (41.3) | 0.025 | 42.3 (55.5) | 26.6 (37.3) | 0.140 |
| INR | 1.3 (0.4) | 1.4 (0.6) | 0.220 | 1.3 (0.6) | 1.4 (0.6) | 0.562 |

Mean (SD) or n (%)
