# Supplementary material for: Right heart failure before LVAD implantation predicts right heart failure after LVAD implantation – is it that easy?
Source: J Cardiothorac Surg. 2020 May 25;15:113. doi: 10.1186/s13019-020-01150-x (PMC7249428; doi:10.1186/s13019-020-01150-x)
Supplement: Supplementary file 2 — Additional file 2: Supplemental Table 2. Risk factors for RHF and death from RHF after one month and one year. [file 13019_2020_1150_MOESM2_ESM.docx]

Supplemental Table 2 Risk factors for RHF and death from RHF after one month and one year

|  | **Combined outcome 30 days** | | | **Combined outcome one year** | | |
| --- | --- | --- | --- | --- | --- | --- |
|  | **RHF** (n=35) | **No RHF** (n=77) | p | **RHF** (n=26) | **No RHF** (n=71) | p |
| Age | 50.4 (14.3) | 55.7 (12.4) | 0.099 | 52.6 (14.3) | 55.3 (12.4) | 0.353 |
| Male | 30 (85.7) | 68 (88.3) | 0.761 | 22 (84.6) | 62 (87.3) | 0.742 |
| **INTERMACS** |  |  |  |  |  |  |
| ▪ 1-2 | 23 (65.7) | 26 (33.8) |  | 14 (53.8) | 25 (35.2) |  |
| ▪ 3-4 | 10 (28.6) | 40 (51.9) |  | 10 (38.5) | 27 (38.0) |  |
| ▪ 5-7 | 2 (5.7) | 21 (27.3) | 0.047 | 2 (7.7) | 19 (26.8) | 0.511 |
| **Indication for LVAD therapy** |  |  |  |  |  |  |
| ▪ BTT | 24 (68.6) | 52 (67.5) |  | 19 (73.1) | 48 (67.6) |  |
| ▪ DT | 8 (22.9) | 23 (29.9) | 0.645 | 5 (19.2) | 21 (29.6) | 0.437 |
| **Underlying disease** |  |  |  |  |  |  |
| ▪ Ischaemic | 12 (34.3) | 27 (35.1) |  | 10 (38.5) | 25 (35.2) |  |
| ▪ Dilatative | 17 (48.6) | 45 (58.4) | 0.822 | 12 (46.2) | 41 (57.7) | 0.437 |
| **Echocardiography** |  |  |  |  |  |  |
| EF <30% | 34 (97.1) | 73 (94.8) | 1.000 | 25 (96.2) | 67 (94.4) | 1.000 |
| Moderate or severe mitral valve regurgitation | 8 (22.9) | 37 (48.1) | 0.793 | 9 (34.6) | 36 (50.7) | 0.334 |
| Moderate or severe Tricuspid valve regurgitation | 7 (20.0) | 31 (40.3) | 0.794 | 10 (38.5) | 28 (39.4) | 1.000 |
| TAPSE [mm] | 14.0 (5.5) | 14.5 (4.6) | 0.746 | 14.2 (5.0) | 14.6 (4.8) | 0.762 |
| VCI diameter | 18.2 (4.9) | 19.4 (4.8) | 0.417 | 19.5 (5.5) | 19.1 (4.6) | 0.774 |
| LAI | 20.8 (9.8) | 27.3 (10.1) | 0.038 | 20.6 (9.7) | 27.6 (10.0) | 0.013 |
| **Right heart catheter** |  |  |  |  |  |  |
| PVR [Dyn*s/cm^5^] | 154 (112) | 238 (163) | 0.143 | 176.0 (143.3) | 245.2 (162.0) | 0.170 |
| CI [L/min*m^2^] | 2.2 (0.6) | 1.9 (0.5) | 0.221 | 2.1 (0.5) | 1.9 (0.5) | 0.287 |
| PAWP [mmHg] | 25.9 (7.8) | 23.6 (11.1) | 0.534 | 21.7 (9.9) | 24.4 (10.8) | 0.391 |
| mPAP [mmHg] | 32.4 (10.1) | 31.6 (13.1) | 0.859 | 28.5 (12.9) | 32.5 (12.3) | 0.286 |
| CVP [mmHg] | 13.0 (5.4) | 10.4 (6.8) | 0.256 | 13.4 (5.6) | 10.0 (6.7) | 0.089 |
| CVP/PAWP | 0.58 (0.29) | 0.44 (0.24) | 0.104 | 0.64 (0.23) | 0.42 (0.24) | 0.005 |
| **Laboratory** |  |  |  |  |  |  |
| Hb [g/dL] | 10.2 (1.7) | 11.7 (2.4) | 0.003 | 10.3 (1.9) | 11.8 (2.3) | 0.003 |
| Leucocytes [Mrd/L] | 8.9 (4.2) | 9.5 (4.9) | 0.593 | 8.9 (5.3) | 9.5 (4.5) | 0.567 |
| Thrombocytes [Mrd/L] | 174 (84) | 214 (91) | 0.073 | 186 (96) | 212 (87) | 0.203 |
| Total bilirubin [mg/dL] | 1.9 (2.1) | 0.9 (0.5) | 0.042 | 1.6 (1.9) | 0.9 (0.5) | 0.062 |
| Urea [mg/dL] | 48.1 (22.6) | 30.1 (15.9) | 0.003 | 42.2 (22.1) | 30.8 (16.6) | 0.023 |
| Creatinine [mg/dl] | 2.4 (1.2) | 1.5 (0.6) | 0.010 | 2.2 (1.1) | 1.5 (0.6) | 0.011 |
| GOT [U/L] | 154.6 (113.5) | 178.9 (113.5) | 0.315 | 73.3 (96.2) | 101.8 (202.5) | 0.493 |
| GPT [U/L] | 195.4 (449.2) | 65.3 (121.0) | 0.215 | 80.7 (141.8) | 95.5 (260.4) | 0.783 |
| GGT [U/L] | 126.3 (75.9) | 127.1 (100.2) | 0.975 | 112.2 (53.7) | 133.0 (106.7) | 0.221 |
| CRP [mg/L] | 28.1 (37.8) | 51.4 (65.3) | 0.139 | 46.0 (60.1) | 27.3 (37.5) | 0.148 |
| INR | 1.2 (0.3) | 1.4 (0.6) | 0.092 | 1.3 (0.6) | 1.4 (0.5) | 0.296 |

Mean (SD) or n (%)
